# Reactive Oxygen Species Enlightened Therapeutic Strategy for Oral and Maxillofacial Diseases—Art of Destruction and Reconstruction

**DOI:** 10.3390/biomedicines10112905

**Published:** 2022-11-11

**Authors:** Yuwei Zhang, Yifei Zhang, Yukun Mei, Rui Zou, Lin Niu, Shaojie Dong

**Affiliations:** 1 Key Laboratory of Shaanxi Province for Craniofacial Precision Medicine Research, College of Stomatology, Xi’an Jiaotong University, Xi’an 710004, China; 2 Clinical Research Center of Shaanxi Province for Dental and Maxillofacial Diseases, College of Stomatology, Xi’an Jiaotong University, Xi’an 710004, China; 3 Department of Prosthodontics, College of Stomatology, Xi’an Jiaotong University, Xi’an 710004, China

**Keywords:** ROS, oral diseases, antioxidant materials, treatment strategy

## Abstract

Reactive oxygen species (ROS) are byproducts of cell metabolism produced by living cells and signal mediators in biological processes. As unstable and highly reactive oxygen-derived molecules, excessive ROS production and defective oxidant clearance, or both, are associated with the pathogenesis of several conditions. Among them, ROS are widely involved in oral and maxillofacial diseases, such as periodontitis, as well as other infectious diseases or chronic inflammation, temporomandibular joint disorders, oral mucosal lesions, trigeminal neuralgia, muscle fatigue, and oral cancer. The purpose of this paper is to outline how ROS contribute to the pathophysiology of oral and maxillofacial regions, with an emphasis on oral infectious diseases represented by periodontitis and mucosal diseases represented by oral ulcers and how to effectively utilize and eliminate ROS in these pathological processes, as well as to review recent research on the potential targets and interventions of cutting-edge antioxidant materials. The PubMed, Web of Science, and Embase databases were searched using the MesH terms “oral and maxillofacial diseases”, “reactive oxygen species”, and “antioxidant materials”. Irrelevant, obsolete, imprecise, and repetitive articles were excluded through screening of titles, abstracts, and eventually full content. The full-text data of the selected articles are, therefore, summarized using selection criteria. While there are various emerging biomaterials used as drugs themselves or delivery systems, more attention was paid to antioxidant drugs with broad application prospects and rigorous prophase animal experimental results.

## 1. Introduction

Redox homeostasis is the basic requirement for biological systems to perform diverse normal cell functions, such as human cell growth, survival, differentiation, and aging. The imbalance between the elimination and generation of reactive oxygen species (ROS) is considered to be the main cause of various human diseases [1]. As a series of normal biological cellular metabolic byproducts and signaling molecules, ROS are mainly produced by the nicotinamide adenine dinucleotide phosphate (NADPH) oxidase in mitochondria, which responds to growth factors and cytokines through physiological signaling pathways [2]. As an essential member of the oxidase superfamily, NADPH can utilize molecular oxygen (O_2_) to produce hydrogen peroxide, hydroxyl free radicals, and other ROS [3]. Furthermore, heat, ultraviolet (UV) light, therapeutic drugs, radiation, and other external factors from the environment can also act as sources of ROS, as showed in Table 1 [4,5,6,7].

Thus, under pathological conditions or external stimulations, cells produce excessive amounts of ROS and ultimately evolve a series of reactions for the fittest survive as responses to the changed environments. As signaling molecules, ROS can upregulate intracellular oxidative stress to activate, following apoptosis, necrosis, autophagy, and a variety of biological processes [14]. Increasing evidence shows that ROS plays a key role in the signaling molecules of the whole cell death pathway. Excessive production of ROS could destroy organelle structure, together with biomolecules, leading to an inflammatory reaction, which is known as a basic mechanism of the induction of cardiovascular, respiratory, and nervous system issues. Besides, end-stage renal failure, diabetes, and cancers are also accompanied by the above ROS-related pathological changes [15,16,17,18,19,20].

The failure of oxidative metabolism may lead to the incongruous consumption and production of reactive oxygen species, resulting in excessive lipid peroxidation and oxidative stress. Mammalian cytochrome P450 (CYP450) is a heme-thiolate enzyme involved in the oxidative metabolism of multifarious exogenous and endogenous lipophilic compounds [21]. P450 has evolved to guard organisms against the toxics as the defense systems. CYP enzymes exist in all tissues, especially with the highest concentration in the small intestines and livers in mammals. These proteins are the kinds of membrane-bound proteins which are abundant in the liver microsome and play a crucial role in the biosynthesis of bile acids and metabolism of xenobiotics, such as environmental pollutants, drugs, and carcinogens. Meanwhile, CYPs are also abundant in the inner mitochondrial membrane of steroidogenic tissues, including the testis, adrenal cortex, ovary, and mammary gland, together with the placenta, and are indispensable in the degradation and synthesis of endogenous steroid hormones. Moreover, CYP enzymes play an important part in cholesterol biosynthesis, unsaturated fatty acid oxidation, and vitamin metabolism. Detailed functions of CYPs in the brain have been demonstrated, including regulating endogenous agonists of gamma absorptiometry aminobutyric acid receptor, eliminating retinoids, and maintaining the homeostasis of brain cholesterol. Therefore, CYPs produce the central effect in the maintenance of cellular homeostasis, as well as cellular metabolism. However, CYP-mediated biotransformation may somehow lead to the metabolic activation of exogenous agents to reactive carcinogenic products, as the process is often referred to ‘lethal synthesis’ [22]. The feckless coupling process of the P450 catalytic cycle leads to continuous ROS generation, which affects the pathways of signaling, together with other cell functions. The production of P450 ROS is strictly controlled by the transcription of gene and the interaction between monooxygenase protein components affecting its activity, coupling, and stability. P450 levels are downregulated by oxidative stress through various feedback mechanisms in turn [23]. Therefore, CYP450 is considered a marker of oxidative stress. It could convert toxic metabolites into superoxides, including hydrogen peroxide, superoxide anions, and hydroxyl radicals, which may induce damage to cells [24].

To neutralize additional ROS, cells have evolved a rather balanced system composed of superoxide dismutase (SOD), glutathione peroxidase (GPxs), catalase (CAT), thioredoxin (TRX), and nonenzymatic antioxidants that jointly reduce the oxidation state [25]. P450 is widely presented in the oral cavity and can participate in oral diseases in response to adverse external stimulation, leading to ‘lethal synthesis’, such as the existence of ROS-related pharyngeal and oral cancer. For example, betel nut is a risk factor for oral cancer, due to arecoline as an initial alkaloid in areca nut, which could be metabolized by prompting the production of ROS through the superfamily of cytochrome P450 (CYP) [26].

As unstable and highly reactive oxygen-derived molecules, free radicals and antioxidant defense act as an indispensable part in the systemic pathological state, significantly in the oral cavity [27]. Studies have proved that monitoring the levels of oxidative stress in saliva may provide a tool for detection, diagnosis, management and treatment of some systemic and oral diseases [28,29]. Some dysfunctions associated with orofacial regions treat ROS as a causal or persistent factor. Related diseases consist of the head and neck cancers associated with tobacco, betel nut, alcohol, genetics, and viruses, including squamous cell carcinoma and melanoma, chronic infectious inflammation (such as caries and periodontal disease), Sjogren’s syndrome, wound repair, mucosal diseases (such as oral ulcers), Behcet’s disease (BD), oral lichen planus and mucosal damage after bleaching, the process of facial aging, maxillofacial manifestations of diabetes mellitus (DM), neurodegenerative diseases (including Alzheimer’s disease), temporomandibular joint syndrome (TMJ), facial pain (such as trigeminal neuralgia), muscle fatigue, and so on. At present, ROS has been confirmed to be closely associated with oral diseases, but there is no systematic review to analyze the pathogenesis and compensation mechanism in the oral and maxillofacial areas. Therefore, this review will probe into the pathogenesis and treatment mechanism of ROS in the oral and maxillofacial areas, in order to re-comprehensively understand the comprehensive role of ROS and make the best use of ROS to prevent and treat oral and maxillofacial diseases. In addition to traditional treatment methods, with the progress of materials science, focuses on the oxidation treatment and anti-oxidation treatment of ROS-related oral and maxillofacial diseases are more compelling. While there are various emerging biomaterials used as drugs or delivery systems, more attention was paid to antioxidant drugs with broad application prospects and rigorous prophase animal experiments.

Many oral diseases are closely related to oxidative stress, such as chronic infections and mucosal diseases. For those which are vulnerable to the damage of free radical during the pathological process, antioxidant treatment strategy is essential. Conventional antioxidants in the treatment of oral diseases are limited by the low concentration without local enrichment, rapid drug metabolism, short residence time at the targeted location, and complex action environment, such as the moist oral environment and deep periodontal pocket, all making it arduous to achieve full doses and promising therapeutic effects. However, with a profoundly in-depth understanding of the disease caused by systemic oxidative stress, many new antioxidants have been introduced into disease treatment. Combined with new drug delivery systems, such as strong adhesive patches, microneedles, temperature-sensitive injectable hydrogels, and so on, more options arise for the treatment of oral and maxillofacial oxidative stress disease. For those that require an oxidizing agent to reach the purpose of treatment, including tooth bleaching, sterilization, disinfection, and the ablation of maxillofacial tumors, the production of ROS seems indispensable [30,31,32,33,34,35,36,37,38,39,40]. Therefore, in the treatment of diseases, it is necessary to choose and balance according to the disease itself, in order to suit the conditions.

## 2. Search Strategy and Record Screen

**Figure 1:**
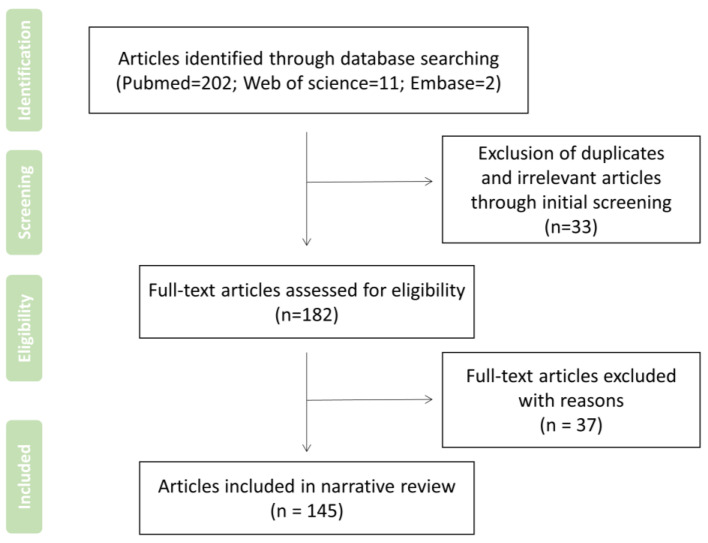
Flow diagram of summarizing search strategy of studies employed in the narrative review.

As a narrative review with no specific need of data aggregation or integration for qualitative or quantitative analysis, the search strategy is also required to be selective, as displayed in Table 2, while the article summarizes what has been written on a subject or topic related to the role of ROS in oral and maxillofacial diseases and the therapeutic significance of the subsequent utilization or elimination of ROS. PubMed, Web of Science, and Embase, up to October 2022, were systematically and selectively searched. Moreover, manual searches and filters proceeded according to the content, reference lists, and related reviews, with an initial comprehensive scope of questions. According to the qualification criteria, first screen the title and abstract, and then screen the whole content. If the article is irrelevant/duplicative to the subject, the experimental design is not rigorous enough, or it is not the latest and is obsolete, and it will be excluded, as illustrated in Figure 1. The research results were independently screened by two authors (Yuwei Zhang and Yifei Zhang). Other authors intervened for consultancies if there were inconsistent opinions.

## 3. ROS and Oral and Maxillofacial Disease-Related Pathological Mechanisms

### 3.1. Oral Mucosal Diseases

Oral mucosal diseases include oral ulcer, BD, oral lichen planus, recurrent aphthous stomatitis, oral pemphigus vulgaris, oral leukoplakia, and others that are featured by autoantibodies or infiltration lymphocytes or intra-epithelial blistering causing interface inflammation and oxidative stress disorder or a loss of cell-matrix adhesion. Clinically, patients with blisters, ulcers, and erosions may affect the skin and the further mucosal surfaces of the eyes, nose, and genitals [41,42]. It has already been stated that free ROS plays a critical role in mucosal damage, such as gastric mucosal damage and oral mucosa damage [43]. Because the oral mucosa allows substances to absorb rapidly, oral cells are particularly vulnerable to the damage of free radical [44]. Studies have pointed out that oxidative stress may be critical to the pathological process of oral lichen planus and oral lichen-like reactions. The increase in saliva and serum malondialdehyde (MDA) in patients with oral lichen planus confirmed their significance as biomarkers of oxidative stress [27]. In vivo research showed that excessive production of ROS in the early stage of oral mucositis and local consumption of a great number of antioxidants, such as glutathione, in oral mucositis may lead to redox imbalance [45], which led to thinking about active treatment strategies. Studies have proven that the drug-taking *Salvia miltiorrhiza* Bunge, as an example, can improve the 2-diphenyl-1-picrylhydrazyl (DPPH)-scavenging ability of cells and block the production of ROS; thereby, the expression of NF-κB and cleaved caspase-3 decreased significantly, followed by the inhibition of cell death. Thus, the proliferation rate of human pharyngeal cells increased and protected the mucosal damage caused by 5-fluorouracil treatment at the same time [46].

To take the actual situation of oral mucosal diseases into account, as one of the typical and common oral mucosal diseases, an oral ulcer, which is also known as a mouth ulcer (MU), the main clinical manifestations were small breakages on the mucosal surface of tongue, gum, inner cheek, or lips. Although an oral ulcer is a very common benign self-curing disease, it will bring an anabatic aching feeling during the activities of daily living, for instance, drinking, brushing teeth, and even talking, which would severely decrease quality of life for its victims. The exact cause of MU still remains unknown, and clinical studies suggest that it is usually due to occlusal trauma, improper dentures, tooth fractures, fillings, genetic, and mental factors. Trauma-related ulcers usually subside within approximately a week after the cause is removed, and patients presenting a mouth ulcer lasting more than three weeks should be prudently referred for biopsy or other investigations to exclude malignancy or other serious conditions [47,48,49]. Significantly, MU is generally accompanied by inflammation, along with high levels of ROS, which deteriorates the patient’s condition [50]. Inflammation and bacterial infection increase the difficulty of MU healing. At present, the treatment of MU is mainly aimed at anti-inflammatory and anti-infection qualities of ulcers to shorten the healing time and reduce symptoms, which are somehow unable to plug up loopholes, in terms of their sources and systems.

Therefore, further scientific research and exploration are indispensable. It has been discovered that vitamin B2-modified iron oxide nanoparticle exhibited the further improvement of catalase-like, peroxidase-like, and SOD-like activities, conducting as a typical iron oxide nanozyme with triad activities. Particularly, VB2 modification remarkably improved SOD-like activity, in order to provide an ROS-scavenging ability, which may be an up-and-coming reagent for the cure of MU [51].

BD is a multisystem autoimmune disease with undetermined etiology that is characterized by chronic recurrent oral genital ulcer and uveitis. Multiple systemic associations were also observed in bipolar disorder, including joint, gastrointestinal, cardiopulmonary, nerve, and vascular involvement [52]. The specific pathogenesis of BD still remains undetermined, however, according to the research, the upregulated-function of neutrophils would lead to phagocytosis, chemotaxis, excessive production of ROS and superoxide anions during the BD process, all these effects may be the cause of oxidative tissue damage in BD. The immune changes of subsets and functions and T lymphocyte abnormalities are considered to be related to the pathogenesis of the disease [53].

The aforementioned research makes the focus begin to convert to the effective elimination of ROS and the restoration of the balance of physiologically active oxygen, which are new insights for the treatment of oral mucosal diseases. According to the discovery and promotion of antioxidants, combined with the appropriate drug carriers suitable for the moist dynamic environment of oral mucosa, innovative treatments for breaking the original symptomatic treatments for a series of complex mucosal diseases with unclear causes, which seemed treacherous before, are now visible.

### 3.2. Periodontitis

**Figure 2:**
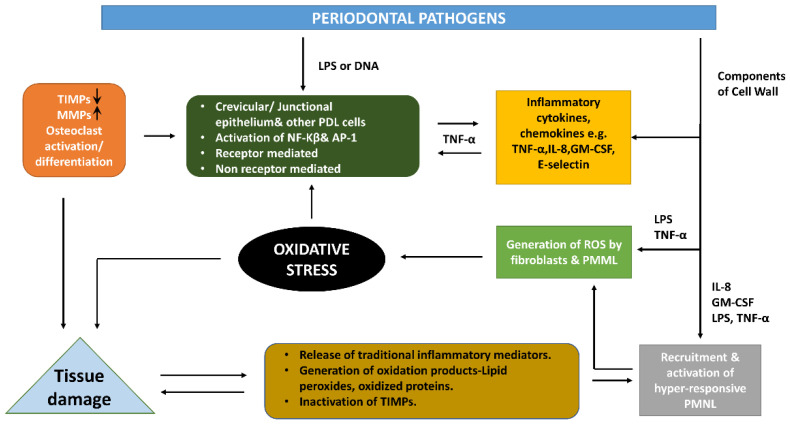
ROS mediated the periodontal pathogen progress of chronic inflammation and the tissue damage, where NF-k β for nuclear factor kappa beta; MMP represents matrix metalloproteinase; TNF for tumor necrosis factor; TIMP for tissue inhibitor of matrix metalloproteinase; IL for interleukin; AP-1 for activating protein-1; LPS for lipopolysaccharide; PDL for periodontal ligament; ROS for reactive oxygen species; GM-CSF for granulocyte-macrophage colony-stimulating factor. The up arrow (↑) indicates the levels of MMPs are up-regulated and vice versa [61].

Periodontal diseases are sorts of diseases, which broadly includes gingival diseases and periodontitis, that, in practical terms, include a wide range of inflammatory conditions that impact the supporting structures of the teeth. Two widely recognized categories of gingival diseases include dental plaque-induced gingivitis and non-dental plaque biofilm-induced gingival diseases [54]. Periodontitis, as another category, is defined according to its severity (chiefly periodontal destruction with symptom of root length and periodontitis-related tooth loss) and complexity of management (intrabody defects, pocket depth, masticatory dysfunction, tooth hypermobility, furcation involvement), and the scope (local or generalized) shall be described addictively to comprehensively evaluate the prognosis [55]. The occurrence and spread of periodontal disease are through the dysregulation of the commensal oral microbiota (dental plaque), which subsequently interacts with the host’s immuno defense, resulting in inflammation and disease. The whole pathophysiological condition will continue to be active and static for a period of time, until the microbial biofilm is detached by treatment or there is no alternative, unless the affected teeth are extracted and the inflammation subsides afterwards. The severity of periodontal disease depends on environmental and host risk factors, both modifiable (e.g., having a smoke) and unalterable risk factors (e.g., genetic susceptibility) [56]. It is acknowledged that dental plaque is a common initiating factor of periodontal diseases. The triggering event of periodontitis is the formation of biofilms in subgingival plaques, due to oxidative stress. However, numerous recent studies have shown that the progression of the disease may be due to the adverse reaction of the immune system to these organisms, which leads to acute inflammation and the release of ROS through polymorphonuclear leukocytes (PMNs) during the phagocytosis of periodontopathic bacteria [57,58]. The imbalance between the ROS produced during this process and existing antioxidants results in oxidative stress, which leads to pathological conditions at the periodontal tissue stage, ultimately ending with heightened oxidative damage to periodontal ligament, gingival tissue, and alveolar bone [59]. In vitro studies have proved that ROS can degrade many extracellular matrix components, such as proteoglycans, leading to the alteration of amino acid functional groups, the fragmentation of core proteins, and limited depolymerization of glycosaminoglycan chains. The identification and characterization of connective tissue metabolites in gingival crevicular fluid (GCF) caused by periodontal tissue, especially alveolar bone degradation, provides further proof for the role of ROS in inflammatory periodontal disease-related tissue destruction [60]. With the existence of periodontal pathogens, tissue destruction interacting with ROS forms an intricate cycle, as illustrated in the simplified diagram in Figure 2 [61]. Meanwhile, it also leads to other diseases.

The prevalence of periodontitis is conspicuously higher among patients with poorly controlled diabetes, and growing evidence unveils oxidative stress in the pathobiology of type 2 diabetes with chronic periodontitis [62,63]. The pro-oxidative state in periodontitis could result in a descent in insulin sensitivity and a remarkably systemic impact impairing organ systems distant from the core of inflammation [64]. The production of SOD antioxidants in diabetic patients decreased. This led to the destruction of redox homeostasis and an increase in wound superoxide free radicals. At the same time, it led to cell injury, hindered cell proliferation, and inhibited wound healing. A study also supported the high levels of ROS production in the peripheral blood cells in pregnant women with gestational diabetes mellitus and emphasized the association with gestational diabetes mellitus [65]. There is also evidence that periodontal disease is associated with other metabolic diseases, cardiovascular diseases, and nervous system diseases through the ROS pathway [66,67,68].

The oral environment mostly belongs to the anaerobic environment. Gram-negative anaerobic or facultative bacteria and anaerobic bacteria are the dominant bacteria in the oral environment within the sub-gingival biofilm. Notably, low redox potential is considered critical to the growth and survival of subgingival anaerobes in the gingival pocket/crevice [69]. Therefore, there is an obvious conflict in formulating a future treatment strategy, based on redox biology, for maintaining a low redox state to protect host cells and tissues from oxidative stress, which is conducive to promoting the growth and survival of anaerobic bacteria. Nevertheless, differ from viruses, bacteria are not all intracellular pathogens, so keeping low redox status in cells may not be relevant to high redox status in gingival pocket/crevice [70].

At present, the treatment strategy of periodontal disease material research and development is still based on two goals: anti-inflammatory and anti-infection. In this process, it reduces the generation of inflammation-related proteins, the formation of osteoclasts, and the production of circulating ROS in patients with periodontitis and promotes the repair and regeneration of periodontal tissue. These outcomes reveal that resveratrol defends rats against periodic tissue damage through curbing inflammatory responses and through stimulating and encouraging antioxidant defense systems [71]. In addition, a randomized controlled trial confirmed that aPDT with 1000 μg/mL toluidine blue O (TBO) and red light-emitting diode (LED) irradiation remarkably inhibited dental plaque formation without harming teeth or the surrounding tissues characterized by the bactericidal effect of Streptococcus oralis, while effectively reducing the quality and quantity of ROS [72]. These findings provide new insights into the blockade of cyclophilin D rescues dexamethasone-induced and mitochondrial dysfunction-related oxidative stress in gingival tissue [73].

### 3.3. Other Chronic Infectious Oral Diseases

Dental caries is a kind of disease with a chronic progressive destruction of dental hard tissue under the influence of many factors dominated by bacteria. Bacterial infection and oxidative stress cause enamel decay and lead to dental caries. The cariogenic bacteria are mainly *Streptococci*, which have been proven to have Spx regulators that play significant parts in the capability to thrive in the mouth with higher oxidative stress [74]. Occasionally, *lactobacilli* adhere to the surface of enamel and dentin to produce a large amount of acid, and the bacterial spectrum changes with changes in the disease state [75,76].

ROS and chronic oxidative stress in the oral cavity, together with acidic pH on the dental enamel surface because of the metabolic activities of bacterial plaque, are the main contributors to the progression and development of dental caries. The buffering capacity and oxidative stress state of saliva are also considered to be promoting factors for the development of dental caries [77,78,79]. Studies have shown that the total antioxidant capacity (TAC) of the saliva of children with dental caries was conspicuously lower than that of children without dental caries [80].

**Figure 3:**
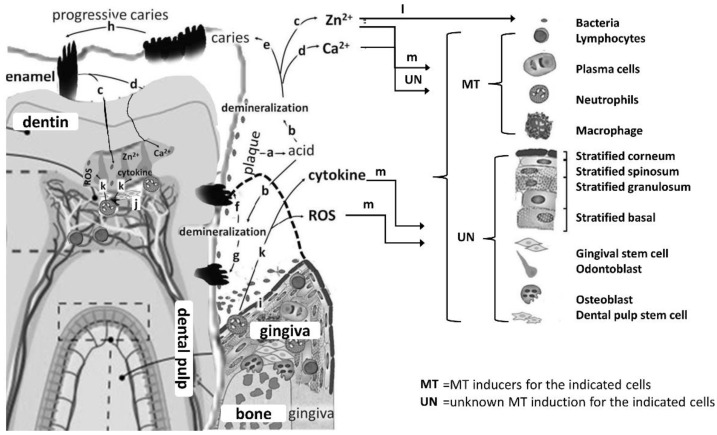
Dental caries versus metallothionein. Bacteria in biofilm produce acid (a) through daily metabolic activities that ultimately could induce demineralization (b), which result in the release of Zn^2+^ (c) and Ca^2+^ (d). The course of demineralization in enamel (e) and dentin (f) leads to the progress of cavitation, as a consequence, especially at susceptible areas of the tooth, such as the occlusal fissures (h) and the root of the tooth (g). Continuous demineralization can lead to cavities and affect the interior of teeth, including dentin. Population of bacteria may also induce infiltration of polymorphonuclear neutrophils and macrophages in the gingiva (i) or dental pulp, in case of (active/progressive) dentine caries (j), which contributes to the gradual releasing of reactive oxygen species (ROS) and inflammatory cytokines (k). ROS (m), Zn^2+^ that is essential for metabolism of bacteria (l) and inflammatory cytokines are notorious for affecting (increased) MT biosynthesis in various nucleated blood cells, including macrophages, neutrophils, and lymphocytes. Nevertheless, the MT biosynthetic potential of odontoblasts, gingival stem cells, dental pulp stem cells, and stratified gingival epithelial cells remains to be determined (unknown). The dotted line indicates the level of healthy gums before gingival retraction [32].

In progressive dentin caries, the bacterial population may also lead to the infiltration of polymorphonuclear neutrophils and macrophages from gingiva or dental pulp, resulting in the release of ROS and inflammatory cytokines. Besides, it is universally acknowledged that ROS and Zn^2+^ inflammatory cytokines can induce metallothionein (MT) biosynthesis, which is a group of small molecular weight (~7 kDa), cysteine-rich proteins in various nucleated blood cells, including macrophages, neutrophils, and lymphocytes, as shown in Figure 3 [32].

In addition to the pathogenic microorganisms mentioned above, Helicobacter pylori can also be planted in the oral mucosa and is usually the dominant bacterium in human gastric tissue. The degree of colonization of the oral mucosa correlated with that of the gastric mucosa [81]. In Helicobacter pylori-infected tissues, infiltrating inflammatory cells produce ROS, which induce mtDNA mutations and cause inflammation by producing various mediators [82,83].

Under infectious conditions, the above-mentioned bacterial species launch the generation of various cytokines, including TNFα, interleukin-8, and circulating PMNs, which produce ROS through respiratory bursts, so as to defend against infection [84]. Countless infectious diseases in humans are closely related to biofilms [85], such as the dental caries and periodontal diseases mentioned above. Moreover, ROS and chronic oxidative stress in the oral cavity and the acidic pH on the enamel surface because of the plaque metabolic activities are the main factors for the occurrence and development of dental caries. However, the formation of free radicals in a short time can degrade the biofilm matrix and quickly eliminate the embedded bacteria. One of the new strategies for controlling plaque biofilms using catalytic nanoparticles with peroxidase-like activity (CAT-NP) has been reported, which can trigger extracellular matrix degradation and lead to bacterial death in the acidic niches of biofilms, leading to dental caries. CAT-NPs with biocompatible Fe_3_O_4_ are used to catalyze hydrogen peroxide to produce free radicals in situ to treat nano catalysts with enzyme-like activity as a method to treat a common biofilm-related oral disease [86].

## 4. Treatment Strategy for Oral Diseases by Encouraging Production or Consumption of ROS

### 4.1. Antioxidant Defense Systems

The anti-oxidation strategy is based on eliminating the adverse effects of oxidative stress on the oral and maxillofacial regions, mainly targeting oral diseases with oxidative stress disorder, especially the negative effects caused by excessive harmful reactive oxygen species, such as the pathological mechanisms of periodontitis, caries, partial mucosal diseases, etc.

An antioxidant is a substance that slows down the decay rate of something, due to oxidation, from which the definition includes enzymatic and nonenzymatic compounds. Endogenous enzymatic antioxidants are well-known for catalases, the glutathione system, and SODs. The SODs, as a category of ubiquitous enzymes, were discovered by Fridovich and McCord in the late 1960s, with the leading conversion from superoxide to hydrogen peroxide or molecular oxygen by the following half-reactions:

M^n^ + SOD^−^ + O_2_^−^ + 2H^+^→M^n+1^ + SOD^−^ + H_2_O_2_

M^n+1^ + SOD^−^ + O_2_^−^→M^n^ + SOD^−^ + O_2_

where M represents a metal; the metal-coordination forms of SOD are Cu^n+^ (n = 1) or Fe^n+^, Ni^n+^ or Mn^n+^ (n = 2) [87,88].

Catalase is an enzyme belonging to the oxidoreductases, which catalyzes the following reactions: $2H_{2}O_{2}\overset{}{\Leftrightarrow }O_{2}+2H_{2}O$.

It is a tetramer composed of four highly compressed polypeptide chains, resulting in the unusual stability of the enzyme. Within the tetramer, four iron groups are presented to allow the enzyme to react with hydrogen peroxide; therefore, it is degraded into two water molecules and one oxygen molecule [89].

The glutathione system consists of GPx and glutathione S-transferase, which play a key role in the detoxification of several electrophilic peroxides and compounds via the reaction catalyzed by glutathione S-transferase [90]. Evidence has proven that cells insufficient of this enzyme will undertake oxidative stress and degenerate at the mitochondrial level [91]. Several classifications of antioxidants are illustrated in Table 3, as follows. 

### 4.2. Advanced Antioxidant Drugs and Their Applications

Under the building of classic antioxidants, various advanced materials have been developed. Next, some new antioxidant materials for ROS, used in fields other than oral and maxillofacial diseases, are introduced to provide new ideas for maxillofacial application.

Astaxanthin, a kind of keto-carotenoid, has a strong antioxidative capability and can effectively scavenge ROS in the human body, which has the strongest antioxidative capacity to defend against lipid peroxidation and interacts with ROS much speedier than protective enzymes, as well [115]. Carotenoids are a kind of organic pigment and presented in photosynthetic organisms or plants, such as some bacterial species and algae [116]. They are auxiliary pigments that allow for the absorption of wavelengths, different from chlorophyll during photosynthesis, and protect chlorophyll from photooxidation, which prevents the influences of unpaired oxygen and forms the ROS produced with the occurrence of sunlight. Recent studies have shown that the anti-inflammatory and antioxidant effects of beta-carotene and astaxanthin may lead to the inhibition of H. pylori-induced inflammation. The growth of H. pylori is repressed by astaxanthin, and beta-carotene inhibits ROS-mediated inflammatory signaling, such as redox-sensitive transcription factors and mitogen-activated protein kinases, while it suppresses the expression of inflammatory mediators, such as inducible nitric oxide synthase, cyclooxygenase-2, and interleukin-8, in the infected areas [117].

Anthocyanins inhibit the generation of ROS in living cells and have been proven to be a potential treatment of gastric antral ulcers by significantly improving the activities of radical-scavenging enzymes, including catalase, superoxide dismutase, and glutathione peroxidase. Furthermore, they can also regulate the activity of matrix metalloproteinase-2 (MMP-2), which plays an essential role in periodontitis and apical periodontitis progression [118,119,120,121,122,123].

Bilirubin, as a cardinal distinctive pigment in bile, has been gradually noted for the treatment of ischemia-reperfusion injury, diabetes, cancer, inflammatory bowel disease, and asthma. Bilirubin has also been proven to eliminate the generation of ROS. Therefore, it has been treated as an effective drug and potent antioxidant nanodrug for the treatment of ROS-mediated diseases [124]. Salvianolic acid B is now one of the major active components of *Salvia miltiorrhiza*, due to its strong antioxidant effect [125].

In recent years, with the rapid development of nanomaterials, various new materials based on gels have changed rapidly. A biocompatible tannic acid (TA)-based nanogel, as a powerful ROS scavenger, is reported in the treatment of further ROS-overproducing inflammatory diseases [126]. Additionally, macrophage-targeting and ROS-responsive polyplexes have been developed to enable the efficient systemic delivery of TNF-α siRNA (siTNF-α) to attenuate hepatic inflammation in mice bearing acute liver failure [127]. TNF-α is also of indelible importance during the development of periodontal disease [128].

In view of the close relationship between the pathological process of diabetes and oral periodontitis, many strategies for eliminating superoxide anion radicals in diabetes are also expected to be applied in the oral area. Considering that high ROS concentrations and recurrent wound infections are difficult to heal, researchers proposed a modified poly(*ε*-caprolactone)-*block*-poly-(glutamic acid) polymer vesicle by simulating the natural SOD to eliminate superoxide anion radicals [129].

Furthermore, concerning the oral field itself, many new biomass materials with confirmed antioxidant properties show the ability to promote oral tissue repair and regeneration. Among them, cordycepin is a promising biological molecule extracted from medicinal mushrooms, which has antioxidant, anti-inflammatory, anti-cancer, and other biological performance. The studies proved the effect of cordycepin in inducing osteogenesis differentiation on human dental pulp-derived stem cells in regenerative medicine. In addition, melatonin, a hormone secreted by the pineal gland of the brain, may become a new antibacterial and antiviral treatment method and approach in the future because of its strong neuroendocrine immune regulation activity and the ability to eliminate free radicals and antioxidants.

### 4.3. Emerging ROS-Enlightened Therapeutic Strategy for Oral Disease

It does not take long to make a profound discovery that, under certain conditions, instead, ROS at a certain concentration, rather low or high, can initiate or participate in many essential signaling and metabolic pathways. This likely explains why systemic, nonspecific antioxidants have failed to apply in the clinic, sometimes with neutral and occasionally even inconsistent detrimental outcomes [130]. A certain degree of reactive oxygen species can also inhibit the reproduction of anaerobic bacteria and facultative anaerobic bacteria.

Photodynamic therapy (PDT) is the photo-treatment of benign or malignant diseases via oxygen, light, and photosensitizing agents, which produces cytotoxic ROS and promotes tumor regression. Many photodynamic treatments have been extensively undergone, and photosensitizers (PSs) are critical for the real-world efficacy, while lasers and oxygen allow for flexible and proper delivery for the cure of diseases. Its mechanism is that, in the occurrence of specific light triggers and oxygen, PS is activated from its ground state to excited singlet, producing ROS and inducing tumor apoptosis. These PSs can be classified by their exclusive active oxygen generation efficiency, absorption wavelength, and chemical structure [131]. The PDT method is broadly used as a therapy for periodontal disease and oral cancer treatment [132,133].

Apart from that, some photothermal materials, such as phototherapeutic antibacterial platforms of polypeptides and copper sulfide nanodots, could produce heat and generate ROS under near-infrared light (NIR). In situ generated heat and ROS, as noncontact-based antimicrobial factors, as well as contact-based antimicrobial peptides, lead to irreversible membrane damage, cell content damage, and bacterial thermal ablation [134].

Moreover, the sterilization mechanism of nonthermal plasma used in stomatology also primarily counts on the production of ROS, ions, electrons, and electromagnetic fields. Currently, due to the safety and stability of nonthermal plasma, it has increasingly become the most potential and innovative treatment in the field of stomatology [135]. In addition, NTP is widely used for sterilization in gingival crevices, biofilm elimination, dental implants, and wound healing, and it can also be used for the treatment of linear gingival erythema, angular stomatitis, and oral candidiasis [136,137].

In the clinical practice of stomatology, lasers can be widely used in tissue repair and surgical resection procedures with reduced pain and tooth sensitivity [138,139]. ROS are activated when exposed to laser treatment. A low concentration of ROS could accelerate the process of growth factors and tissue restoration, while excessive ROS may impair the basic protein components of cells. Therefore, an appropriate laser exposure time and intensity are key in the process of dental treatment. Cold laser therapy is a common low-level infrared light therapy. Moreover, laser-generated ROS is an effective method to heal dental pulp wounds and promote the formation of restorative dentin [140,141,142].

In the process of tooth whitening, various oxidants are used to bleach teeth. Carbamide peroxide, hydrogen peroxide, sodium hypochlorite, ozone assisted by titanium dioxide, and hydroxyapatite nanoparticles are common oxidants used in the process of tooth bleaching. In the process of oxidation, they can produce ROS, penetrate dentin, decompose organic matter, and achieve cleaning and sterilization. At the current stage, the potential side effects of tooth whitening, such as the toxicity of whitening agents, soft tissue injuries, and adverse effects on the hardness and roughness of human enamel, are inevitable [143,144,145].

## 5. Summary and Outlook

In conclusion, ROS are produced under various inner or outer stimulations, such as cytokines, growth factors, chemokines, shear stress, and hypoxia. In addition, several important signal cascades or biological pathways are regulated by ROS, including MAPK, GPCR, Notch, Wnt-*β*-catenin, NF κB, JAK-STAT, and PI3K/AKT.

ROS contribute to various pathophysiology activities of the oral and maxillofacial regions and participate in the benign oral lesions and multiple stages of carcinogenesis. Especially in head and neck cancers, assorted common risk factors lead to cancer through the mechanism of ROS, including tobacco, betel nut, alcohol, and virus. In view of the extensive effects of ROS on carcinogenesis, ROS and their related pathways are attractive intervention targets. At present, many ROS-sensitive polymeric nanocarriers for synergistic cancer therapy have been developed.

The treatment strategies of oral and maxillofacial ROS-related diseases are mainly anti-inflammatory and anti-infection. On the one hand, this strategy helps to control the microbial infection of the moist oral cavity; on the other hand, it aims at the adverse inflammatory effect caused by the surplus of ROS activated by macrophages, which is caused by microbial infection and other reasons. Overall, ROS-mediated oxidative stress diseases are reflected in all systems of the body. In the future, we should focus on the integration of multidisciplinary and multisystem knowledge and technology to address the diseases induced by the imbalance of ROS. Of particular concern is the manifestation of systemic oxidative stress in the oral cavity, such as the oral manifestations associated with diabetes. In recent years, studies regarding the periodontitis, osseointegration, or bioadaptation of implants to oxidative stress and the mechanism of oxidative stress, in relation to pathological conditions, including diabetes, have brought new development foreground for the treatment of ROS-related diseases.

The renewal of drugs inspires a comprehensive view of the biosphere and new antioxidants and oxidants for the treatment of human systemic oxidative stress disorders. Focusing on the special physiological results of oral and maxillofacial regions, reasonable drugs and appropriate carriers are equally important. The interior of the oral cavity is a moist, saliva-washed environment, which shortens the local residence time of the drug, as the target concentration could not be enough. In addition, the complex pathological environment weakens its effect, for instance, deep periodontal pockets and endodontic lesions make it difficult to effectively use the drug locally. Therefore, according to the corresponding environment, it is necessary to develop further materials with high adhesion energy and strength for oral soft tissues. Materials with good multiphase phase transition are more suitable for complex and changeable deep pathological cavities, for example, temperature sensitivity with mouth-temperature gelling type, multifunctional crosslinking, and functional modified injectable hydrogel own outstanding contributions to carrying ROS-corresponding drugs in oral cavity. For the overall view of oral and maxillofacial areas occupying the main part of facial region, it is necessary to pay attention to the treatment and aesthetic needs of the integration of repair and regeneration. Combined with the above, it can be regarded as a new drug development and treatment strategy for oral and maxillofacial oxidative stress diseases.

To be fair, ROS is not necessarily a single injury factor. Unlike the data aggregation or integration sort of systematic review, meta-analysis, or other quantitative synthesis research, this review is more inclined to accepted theories, reviews, in vitro studies, and frontier animal experimental research, without the need for explicit study selection or quality appraisal, making some bias inevitable. The regulation of ROS levels is more likely to be the balance of the aesthetic art of destruction and reconstruction in the human body. The basis for the construction of new biomaterials in the future is to balance the amount of ROS and skillfully use or eliminate ROS to terminate the pathological state of the human body and reconstruct a healthy physiological state.

## Figures and Tables

**Table 1:** External factors from the environment as sources of ROS.

External Factors	Mechanism	References
Heat	Atrocious heat exposure induces the damage of mitochondria and causes excessive production of mitochondrial ROS, which can upregulate the transcription of heat shock factors (HSFs) and heat shock proteins (HSPs).	[8]
Ultraviolet light	Generally, UV can directly affect cell components or photosensitive mechanisms to promote the production of ROS. To be specific, UV can affect catalase process and upregulate the synthesis of nitric oxide synthase (NOS), which leads to decreased expression of protein kinase C (PKC) and increased production of ROS.	[9]
Pharmaceutical products and cosmetics	Some therapeutic drugs or cosmetics, such as the photochemical interaction with light, photosensitizer (PS), and molecular oxygen, produce ROS, contributing to oxidative stress, genotoxicity, inflammation, potentially carcinogenesis, and metabolic change, which eventually brings about the apoptosis of cells.	[10,11]
Other radiation	Besides UV-A, UV-B, or thermal radiation, X-ray exposures and other radiation could also generate ROS, which may be due to the observed oxidative stress.	[12,13]

**Table 2:** Search strategy.

PubMed:	Web of Science	Embase
((((“Oral diseases”[Mesh] OR “Maxillofacial diseases”[Mesh]) OR “Oral cavity”[Mesh]) OR “Oral and maxillofacial”[Mesh]) OR (((((((oral mucosal diseases[Title/Abstract] OR periodontitis[Title/Abstract]) OR periodontal disease[Title/Abstract]) OR dental caries[Title/Abstract]) OR periodontal therapy[Title/Abstract]) OR Oral antioxidant treatment[Title/Abstract]) OR antioxidant drugs[Title/Abstract]) OR oral diseases therapy [Title/Abstract]) AND ((((“ROS”[Title/Abstract] OR reactive oxygen species[Title/Abstract]) OR anti-ROS[Title/Abstract]) OR oxidative stress[Title/Abstract]) OR ((oxidation treatment[Mesh] OR pathological mechanism[Mesh]) OR “antioxidant ”[Mesh]))	#1:TS = (oral diseases) OR TS = (maxillofacial diseases) OR TS = (periodontal diseases) OR TS = (oral mucosal disease) OR TS = (periodontitis) OR TS = (dental caries) OR TS = (periodontal pocket) OR TS = (oral diseases therapy) #2:TS = (ROS) OR TS = (reactive oxygen species) OR TS = (oxidative stress) OR TS = (antioxidant) OR TS = (antioxidant material) #1 AND #2	‘oral diseases’/exp OR ‘maxillofacial diseases’/exp OR ‘periodontal disease’/ exp OR ‘periodontitis’:ab,ti OR ‘dental caries’: ab,ti OR ‘oral mucosal disease’: ab,ti OR ‘periodontal tissue’: ab,ti OR ‘oral diseases therapy’: ab,ti OR ‘periodontal treatment’: ab,ti AND (‘reactive oxygen species’/exp OR ‘ROS’/exp OR ‘oxidative stress’/exp OR ‘leptin’: ab,ti OR ‘antioxidant’: ab,ti OR ‘antioxidant treament’: ab,ti OR ‘antioxidant material’: ab,ti OR ‘oxidation treatment’: ab,ti)

**Table 3:** Examples of key antioxidants classified by different criteria.

Classification Criteria	Classifications	Examples	References
Functional mechanism	Preventative antioxidants	DNA repair enzymes, catalase, superoxide dismutase enzymes, glutathione peroxidase superfamily, e.g., selenium-containing glutathione peroxidases (GPxs).	[92,93,94,95,96,97]
		Metal ion sequestrators aiming at metal-induced toxicity and carcinogenicity: albumin, lactoferrin, transferrin, haptoglobin, ceruloplasmin, Vitamin E, melatonin, ascorbate, carotenoids, hemopexin, catalase, superoxide dismutase, polyphenolic flavonoids, glutathione peroxidase, uric acid, glutathione reductase.	[96,97]
	Radical scavengers or chain-breaking antioxidants	Carotenoids, such as vitamin A, α-tocopherol (vitamin E), ascorbate, catechol, edaravone, melatonin and tryptophan derivatives and other thiols (free or protein bound), uric acid, polyphenols (flavonoids), albumin, bilirubin, ubiquinone (reduced form), reduced glutathione.	[98,99]
Location	Intracellular	Mainly hydrophilic scavengers, including catalase, DNA repair enzymes, superoxide dismutase enzymes 1 and 2, ascorbate, glutathione peroxidase, ergothioneine.	[100]
	Extracellular	Ascorbate, selenium-glutathione peroxidase, superoxide dismutase enzyme 3, albumin, lactoferrin, transferrin, uric acid, haptoglobin, ceruloplasmin, carotenoids, e.g., astaxanthin and allopurinol.	[88,101,102,103]
	Membrane associated	Hydrophobic scavengers are presented in cell membranes, where they restrain or interrupt chain reactions of lipid peroxidation, such as α-tocopherol (vitamin E), carotenoids, deoxyanthocyanins.	[100,104]
Protected structure	DNA protective antioxidants	DNA-repairing enzymes, including photolyase, endonuclease and 8-oxoguanine glycosylase, reduced glutathione, superoxide dismutase enzymes 1 and 2, cysteine and glutathione peroxidase.	[93,105,106,107]
	Protein-protective antioxidants	Sequestration of transition metals by preventative antioxidants. Antioxidant enzymes as stated above. Scavenging by the competitive reaction scheme, competing substrates.	[96,97,107,108]
	Lipid-protective antioxidants	Ascorbate (vitamin C), α-tocopherol (vitamin E), reduced ubiquinone, carotenoids, reduced glutathione, bilirubin, and glutathione peroxidase.	[100,104,109]
Origin	Exogenous antioxidants (obtained simply by the diet): phytonutrients	Carotenoids (e.g., astaxanthin), ascorbic acid, tocopherols (α, β, γ, δ), polyphenols (resveratrol, theaflavins, thearubigins, flavonoids, catechins), folic acid, cysteine.	[71,110,111]
	Endogenous antioxidants (synthesized by the body)	Hemocyanin, superoxide dismutase, ferritin, catalase, glutathione peroxidase, reduced glutathione, glutathione S-transferase, ceruloplasmin, proteases, transferrin, peroxisomes, glycosylases.	[96,97,112]
	Artificial synthetic	Aspirin, statins, and renin-angiotensin system inhibitors, N-acetylcysteine, penicillin amine, tetracyclines.	[113,114]

## Data Availability

Not applicable.
